# What do patients with unmet medical needs want? A qualitative study of patients’ views and experiences with expanded access to unapproved, investigational treatments in the Netherlands

**DOI:** 10.1186/s12910-019-0420-8

**Published:** 2019-11-09

**Authors:** Eline M. Bunnik, Nikkie Aarts

**Affiliations:** Department of Medical Ethics and Philosophy of Medicine, Erasmus MC, University Medical Centre Rotterdam, Wytemaweg 80, 3015 CN Rotterdam, The Netherlands

**Keywords:** Compassionate use, Expanded access, Investigational drugs, Patients’ attitudes, Ethical issues, Qualitative study, Interviews, Focus groups

## Abstract

**Background:**

Patients with unmet medical needs sometimes resort to non-standard treatment options, including the use of unapproved, investigational drugs in the context of clinical trials, compassionate use or named-patient programs. The views and experiences of patients with unmet medical needs regarding unapproved, investigational drugs have not yet been examined empirically.

**Methods:**

In this qualitative study, exploratory interviews and focus groups were held with patients with chronic or life-threatening diseases (*n* = 39), about topics related to non-standard treatment options, such as the search for non-standard treatment options, patients’ views of the moral obligations of doctors, and the conditions under which they would or would not wish to use non-standard treatment options, including expanded access to unapproved, investigational drugs.

**Results:**

Respondents had very little knowledge about and/or experience with existing opportunities for expanded access to investigational drugs, although some respondents were actively looking for non-standard treatment options. They had high expectations of their treating physicians, assuming them to be aware of non-standard treatment options, including clinical trials elsewhere and expanded access programs, and assuming that they would inform their patients about such options. Respondents carefully weighed the risks and potential benefits of pursuing expanded access, citing concerns related to the scientific evidence of the safety and efficacy of the drug, side effects, drug-drug interactions, and the maintaining of good quality of life. Respondents stressed the importance of education and assertiveness to obtain access to good-quality health care, and were willing to pay out of pocket for investigational drugs. Patients expressed concerns about equal access to new and/or non-standard treatment options.

**Conclusion:**

When the end of a standard treatment trajectory comes into view, patients may prefer that treating physicians discuss non-standard treatment options with them, including opportunities for expanded access to unapproved, investigational drugs. Although our respondents had varying levels of understanding of expanded access programs, they seemed capable of making well-considered choices with regard to non-standard treatment options and had realistic expectations with regard to the safety and efficacy of such options. Dutch patients might be less likely to fall prey to false hope than often presumed.

## Background

Patients have unmet medical needs when adequate treatment options are not – or no longer – available for their condition. Sometimes, patients with unmet medical needs may wish to look beyond standard treatment options, and may be eligible to try investigational drugs: new drugs that are not yet approved for marketing and are still under investigation. Access to investigational drugs will usually be possible only in the context of a clinical trial. However, when seriously ill patients who have exhausted standard treatment options^1^ cannot be enrolled in clinical trials, their doctors might consider ‘expanded access’ to investigational drugs.

In many countries, expanded access can be requested for individual patients, through so-called ‘named-patient’ programs, or for groups of patients, in so-called ‘compassionate use’ programs. The latter are often initiated by pharmaceutical companies after the successful completion of phase III clinical trials, to bridge the gap between the trial and the commercial launch of the new drug. The company usually supplies the drug for free. Individual- or named-patient expanded access is often initiated by treating physicians in ‘back against the wall’ situations. The physician will need to ask the pharmaceutical company to release the investigational agent, and to request a drug regulatory authority to approve the request for ‘named-patient’ usage of the unapproved drug. Through named-patient programs, drugs may be provided earlier in the drug development process, after completion of phase I or IIa clinical trials. In some countries, including the United States of America (USA), Spain and Italy, and in some hospitals in the United Kingdom, approval from a research ethics review committee or institutional review board is mandatory [1, 2]. In countries where expanded access is not reimbursed, the uptake is generally very low.

It is believed that health literate, high-income and well-connected patients are likelier to succeed in obtaining access to investigational drug outside of clinical trials than other patients [1, 3]. In many countries, few physicians actively engage with expanded access, which likely results from both ethical concerns and the many hurdles that must be overcome in order to obtain investigational drugs: the paperwork, the time, funding and the uncertainty regarding the outcome [2]. Consequently, many doctors are not aware of opportunities for expanded access or have little personal experience with it [4], and may not bring it up as a possibility when their patients run out of standard treatment options. It is estimated that doctors submit around a thousand requests for expanded access for individual patients to the Food and Drug Administration (FDA) in the USA annually [5], for instance, and between 100 and 200 requests annually in the Netherlands [6], although it should be noted that based on one approved request, doctors may prescribe the treatment for multiple (similar) patients.

Various groups around the world, however, are trying to change this, by raising awareness of expanded access and/or facilitating its processes. In the USA, for instance, the Right-To-Try group has successfully campaigned for a federal law allowing seriously ill patients to use unapproved drugs without the need for authorization by the FDA [7]. While this legislation has been heavily criticized by legal experts and bioethicists alike [8, 9], its popularity signals public support for the general idea that patients should be allowed to access unapproved drugs if nothing else is available. Furthermore, as patients and patient advocacy groups can now easily access scientific publications online, they are becoming more knowledgeable about drug development activities around the world. Drug development usually takes a long time [10], and patients are not always patient. In Europe, after a drug has been approved centrally by the European Medicines Agency (EMA), it may not be available within the healthcare system until national authorities have made reimbursement and pricing decisions, which can take up to a year [11]. Patients who have exhausted standard treatment options and are facing life-threatening illness may wish to access the new drug sooner. As a result, demand among patients for expanded access (through named-patient or compassionate use programs) may be rising [5].

These developments have raised ethical issues on various levels, which have been discussed elsewhere [12, 13]. With regard to individual patients, the most pertinent ethical issues include the uncertainties regarding the safety and efficacy of the incompletely tested drug, the vulnerable position that patients with unmet medical needs may find themselves in, concerns related to the exploitation of patients’ hopes and the associated difficulties of obtaining informed consent. These issues are often brought up by experts, however, not patients themselves.

To our knowledge, no empirical research has thus far been directed at patients’ views of - and experiences with - expanded access. Little is known about patients’ awareness of opportunities for access to unapproved, investigational treatment options, their motivations for pursuing non-standard treatment options or not pursuing such options, or the obstacles encountered by patients in the process of seeking information about non-standard treatment options, talking about such options with their treating physicians, or requesting access. In the context of this qualitative study, interviews and focus groups have been conducted with Dutch patients suffering from serious chronic or life-threatening diseases to learn about their experiences - if any - with expanded access to unapproved, investigational drugs, and about their concerns, preferences and expectations with regard to non-standard treatment options, when the end of a standard - curative or life prolonging - treatment trajectory comes into view.

In the Netherlands, the same set of formal condition applies to expanded access as in many other health care systems: patients must suffer from serious and/or life-threatening illness, must have exhausted standard treatment options, and must not be eligible for participation in clinical trials. There are two distinct routes to expanded access: 1) individual requests for the named-patient program, for which treating physicians usually collaborate with hospital-based pharmacists, and which are evaluated by the Health Inspectorate, and; 2) compassionate use programs, initiated by pharmaceutical companies, to which treating physicians apply directly for release of the drug for individual patients or groups of patients, and which are evaluated by the Dutch Medicines Evaluation Board. In the Netherlands, hospitals do not usually accept out-of-pocket payment from patients and usually and offer medical care that is covered by health insurance. Thus, expanded access can generally only take place if the pharmaceutical company supplies the investigational agent at no cost, if the hospital is willing to pay for the drug or (in rare cases) if a health insurer is lenient and willing – by exception – to cover the costs of the treatment. The uptake of the named-patient program is an estimated 100–200 requests annually in a population of over 17 million. Evaluation by a research ethics review committee or institutional review board is not required in the Netherlands. Although Dutch regulatory routes to expanded access differ in some respects from those in other countries, many of the themes discussed are of relevance to systems for expanded access in other countries, as well.

## Methods

To map patients’ experiences with and views of expanded access to investigational, unapproved drugs, we held exploratory interviews and focus groups with patients with potentially unmet medical needs. The Research Ethics Review Committee of Erasmus MC evaluated the protocol and provided a waiver (MEC-2016-275). All interviews and focus groups were held in Dutch.

### Focus groups

**Fig. 1:**
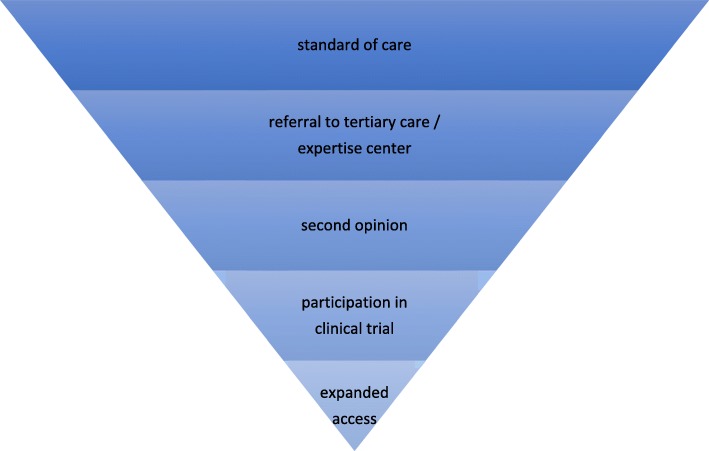
The various stages patients may journey through after they run out of standard treatment options

In total, five focus groups were conducted, each with 6–7 participants (in total: 34 participants), four focus groups with patients who were representative of the general patient population in the Netherlands and one focus group with ‘expert patients’. As we expected, based on the literature, that patients’ experiences with expanded access would be limited, we developed an interview guide (see Additional file 1) in which we successively discussed standard of care and to what extent patients were aware of a standard of care and actively sought information about standard and non-standard treatment options. We discussed steps that are taken when standard treatment options are no longer available or do no longer suffice: referrals to medical specialists working in tertiary care or centers of expertise within university medical centers, second opinions, participation in clinical trials, and expanded access. Thus we discussed the various more or less chronological stages in which patients would be confronted with standard or non-standard treatment options [14, 15], converging toward the use of unapproved drugs (see Fig. 1). We anticipated that fewer and fewer participants would have personal experience with each consecutive stage, like a funnel. Patient journeys may also follow different routes, but during the focus groups, we used this funnel as a directive guide.

Alongside this funnel, patients may seek or be confronted with non-standard treatment options such as alternative and complementary treatments or ‘experimental’ therapies offered through questionable for-profit providers in foreign countries. During our focus groups, we made clear to respondents that such treatment options were not within the scope of our discussion.

Through open-ended questions, we encouraged patients to put forward experiences, views or narratives that they themselves considered important with regard to access to (non-standard) medical treatment. When we discussed the topic of expanded access, we asked participants to consider questions in silence and write down their answers on a piece of paper (i.e. ‘Under what conditions would you consider trying an unapproved, investigational drug?’, ‘What would be reasons for you to try (or to *not* try) a new drug?), before we continued with the discussion. When we asked respondents under what conditions they would be willing to try an investigational drug, we explained clearly that we were talking about drugs under clinical investigation, for which there was some - but not sufficient - evidence of safety and efficacy, and which have not been approved for marketing.

For the first four focus groups, we asked a recruitment agency (CG Selecties) to invite participants from a representative sample of Dutch inhabitants of the Rotterdam region and the Amsterdam region, including surrounding smaller towns and cities. We held two focus groups in Rotterdam at Erasmus MC in September 2016, and two focus groups in Amsterdam in a public library in October 2016. Two focus groups were held in the afternoon, and two focus groups were held in the evening hours. All focus groups lasted 2 h at minimum. For each focus group, 7 participants were invited. The invitation stated that the researchers were looking for people suffering from a chronic and/or serious disease or condition for which they use or have used medications. Participants were offered 50 euros to cover their travel costs and other expenses. These focus groups are from now on referred to as F1 to F4 (focus groups 1 to 4) when quoted.

The fifth focus group comprised six respondents who were patients, but also active members or founders of patient organizations, and can be thought of as ‘expert patients’. ‘Expert patients’ have been defined as patients “who (have) been empowered with the skills, confidence and knowledge needed to play an active role in making decisions about their own health care and management of their chronic condition” [16]. Expert patients are both active participants in the self-management of their disease and health literate. Therefore, they are not representative of the general patient population, where health literacy may be more limited [17]. The focus group was held at Erasmus MC in July 2016, and lasted 2 h. Respondents were recruited through a representative of the Dutch Federation of Cancer Patients (NFK), who was involved in the research project as a stakeholder. When presenting results from this focus group, it is referred to as FE (focus group experts).

### Interviews

Semi-structured interviews were held with 5 individual patients, who were sampled purposively either through members of our valorization panel^2^ or through other contacts. We actively sought patients who may have had personal experiences with expanded access. The same interview guide was used for the interviews. The interviews were meant to yield personal narratives and more in-depth insight in the experiences of patients who (may) have used unapproved drugs. It should be noted that most of these patients should also be considered experts patients. Patients were Dutch and from the same or similar regions in the Netherlands as F1–4 respondents. Interviews were conducted over Skype, at Erasmus MC or at patients’ homes, and lasted between 1 and 2 h. When quoted, the interviews are referred to as I1 to I5 (interviews 1 to 5).

### Analyses

Focus groups and interviews were audio-recorded and integrally transcribed verbatim. NVivo 10 and 11 were used as tools to systematically code the data. The authors (EB, NA) independently coded (the same) three transcripts and developed codebooks by creating, grouping and re-grouping codes into hierarchies or themes. Coding and codebooks were compared and differences were discussed and resolved. After EB coded more transcripts, the researchers again independently coded a transcript and discussed and resolved any differences. Remaining transcripts were coded by EB, and were all thematically analyzed to provide qualitative description [18] of patients’ views and experiences. Quotes were selected and translated to English by EB.

## Results

### Respondents

The focus groups in Rotterdam were attended by 14 participants in total (13 women, 1 man), whose ages ranged from 22 to 63 (median age: 50). In Amsterdam, the focus groups were attended by 14 participants (7 men, 7 women), whose ages ranged from 19 to 79 (median age: 62,5). Eighteen participants had received lower or higher secondary education, 8 had received higher vocational education, and 2 had attended universities. The majority of participants did not have paid jobs (*n* = 23): some were declared unfit for work (*n* = 6), looking for employment (*n* = 3), or retired (*n* = 6). Some participants did voluntary work, and one was a student. Five participants had paid jobs. Some participants lived in the city, some in smaller towns in the region.

All participants used or had used pharmaceutical medications. Patients suffered from cancer of the brain, skin, breast, bladder or larynx, neuromuscular disorders (multiple sclerosis, myasthenia gravis, epilepsy), rare diseases or conditions (cryoglobulinemia, Addison’s disease, Congenital Ichthyosiform Erythroderma) or chronic diseases or conditions (chronic obstructive pulmonary disease, diabetes mellitus, rheumatism, rheumatoid arthritis, Crohn’s disease, hypothyroidism, epilepsy, cardiac arrhythmia, ocular herpes, auto-immune hepatitis, familial hypercholesterolemia, chronic depression, fibromyalgia). Many participants had multiple conditions.

In our focus group with expert patients, some participants underlined at the outset that they spoke not only about themselves, but also on behalf of dozens or hundreds of other patients. Some indicated that they differed from ‘the average patient’ in many respects, including knowledge level and understanding of the disease and treatment options, and the ability to cope psychologically with their (past) disease. Three patients were female, three were male. All patients were oncology patients, having been diagnosed with (metastasized) colorectal, breast, prostate and pharyngeal cancer, neuroendocrine tumors and melanoma. Respondents of our individual interviews ranged in age between 46 and 66, two were male and three were female. Patients had been treated for breast cancer (2 respondents), colon cancer, prostate cancer or renal cancer. Respondents of F1–4 did not have first-hand experiences with expanded access. Some respondents of FE and individual interviews did have first-hand experience with expanded access.

### Themes

Six key themes were identified based on our discussions with patients about non-standard treatment options, and will be presented below: experiences with standard of care, patient assertiveness, the patient’s search for other options, reasons (not) to pursue non-standard treatment options, patients’ expectations of physicians with regard to other non-standard options, and reimbursement of non-standard treatment options. The themes are presented in a roughly (chrono) logical order following the scheme in Fig. 1. In all themes, the doctor-patient relationship plays a prominent role.

#### Experiences with standard of care

The topic of access to medical care elicited immediate reactions in focus group participants, which, we observed, many of them had a desire to share. Although we did not ask any specific questions about this topic, in all five focus groups, respondents spontaneously offered narratives about medical mistakes and mishaps. Diagnoses were missed, including fractures, solid cancers, infections, and appendicitis; mammography images had got lost; doctors had continued with off-label treatment of an autoimmune disease despite serious side effects; a major operation had to be postponed last-minute because of a pre-screening error; one patient suffered from serious long-term neuropathy problems as a result of a preventable complication; a skin lesion was removed by a befriended primary care surgeon but not sent in for pathological examination, and later turned out to be melanoma, by then metastasized; and artifacts on MRI or CT that later proved not to be metastases, implying that the patient was not going to die after all. Despite these negative experiences, respondents expressed a certain degree of understanding and acceptance; health professionals, like other human beings, make mistakes. This was thought of as normal. Doctors have to be jacks-of-all-trades, it was felt: when they must excel at diagnosis and surgery or pharmaceutical treatment *and* communication with patients, they will be fallible at times. Respondents did not usually issue any formal complaints or take any legal steps:

*So afterwards I did send a letter to the board of the hospital. And I wrote: this is not a complaint. This is a suggestion for improvement. Because I think it is too easy just to issue complaints. These people, they are trying to do the right thing, that is what I expect. And they all have their professional ethics; they are trying to help patients get better. And things do go wrong. But where in society don’t things go wrong? I think you have to try constructively to guide them. Not by complaining, but by showing them how you would like to have things. (FE).*

Although we, likewise, did not intend to elicit respondents’ appraisals of the attitudes of their treating physicians, focus group discussions did tend to take this turn and centered around patients’ views of the doctor-patient relationship. Overall, respondents were positive about their doctors. Patients valued a helpful and guiding attitude in their primary care physicians, and liked it when their doctors gave them the feeling that they had “all the time in the world” (F1), that they had the time to explain things and to talk to patients. Respondents wanted their doctors to show compassion, to see and treat them like human beings. Also, they wanted physicians to be accessible, through email or telephone. One respondent mentioned, for instance, how she appreciated that she could always pick up the phone and if needed, she could go see her doctor “within 15 minutes” (F3). Another patient spoke with gratitude of a doctor who, one night, had remained with him and his dying wife until four o’clock in the morning (F3). Patients also valued honesty and truthfulness, and appreciated it when doctors showed that they, too, had their limitations and were not omniscient:

*I am lucky, because I hear a lot of horror stories from people around me about their primary care physicians, but I am lucky because mine is always collaborative. And if I come with something, he will, together with me, look at it seriously. And he’ll say: ‘I don’t know, I’m going to find out.. and sometimes I am even there in the room, and he’ll call a medical specialist to check something. Or he’ll just grab a book to look something up. (F1).*

*So I see this internist and he tells me what I’ve got. And he says: ‘I don’t know anything about this disease. I’ve been looking it up,’ he says, ‘but I’ll go and learn about it’. And he’s been in contact a lot with doctors in the USA. And this guy has been learning about this condition.. Well, he is now the only one in the Netherlands who knows exactly what it is. He has really been absorbed by the whole story. That’s why I’m so happy with him. (F3).*

Some respondents, however, were negative about some of their doctors’ attitudes. In their experience, many doctors did not have time to talk, to listen or to explain things, and would only have 5 or 10 min for their patients. Respondents also felt that doctors were not always equipped with social or communicative skills, showed little interest in their patients’ experiences, had trouble making contact, and did not understand that not all patients were the same. Some doctors were impolite, blunt or hurtful:

*So I go see the surgeon who had done the operation and he says: ‘You’ve got cancer’. And then he turns to the assistant and says: ‘Will you take it from here? Help these people to a glass of water. Because I have to continue with my consultations.’ That was my health care. (FE).*

*Well, it is the nurses who do all the work, putting that thing in your bladder, and the doctor is running from one room to the next. And then he’s gone. You can never ask him anything.. Get him to explain anything? No. (F3).*

At times, negative experiences with treating physicians were a reason for respondents to start looking for other options, and seek referral or second opinion within the Netherlands, or even treatment abroad. Patients’ journeys towards expanded access start against the backdrop of their experiences with standard of care.

#### Patient assertiveness

Respondents felt that, often, at their hospitals, they were not able to access other treatment options than standard of care: they had to take initiative to get a referral or a second opinion and/or they had to seek information about clinical trials themselves, for their doctors would not mention trials. Respondents felt that it is important for patients to learn about their own conditions, to increase their health literacy, to take steps to inform and empower themselves. Patients need to be alert for medical errors or negligence, and to be assertive and ask for diagnostic tests and/or best available treatment options. Respondents stressed that they had to argue and to push for what they wanted or needed:

*So I ended up in a cast for half a year, and that has taught me to just be so, so enormously alert to the signals within your body. And to just not accept whatever it is that doctors are telling you. If you think ‘this is wrong’, well, you have to push on. (F2).*

*By now [medical erros] have happened to me so many times that I am not taking any nonsense anymore. I will tell [healthcare professionals] exactly what I feel and what I think, especially now with this melanoma. If I see a doctor and am told ‘Oh, no, probably nothing is wrong’, I now tell them: ‘Please take a biopsy of this lesion’. That’s it. And I don’t want to go and sit in any doctor’s chair, but if I ever get another medical error… (F1).*

Also, patients reported that doctors sometimes suggested clinical management that they did not agree with, and that they experienced little freedom to depart from standard of care. Many respondents were aware of protocols or standard treatment trajectories, and felt that physicians directed them towards or within those protocols in ways that did not align with their own preferences and treatment objectives:

*R. Because they want you in a trajectory, it happened to me, too, because you are a number and you have to be in this trajectory. […].*

*R. Because of this breast cancer, you enter a trajectory. And that means that you get chemotherapy, and after that radiotherapy.*

*R. Right.*

*R. And then you get a conversation and I said: ‘No, I don’t want that.’ At first they were very friendly and they gave me a week to think it over, and I said: ‘No, I don’t want chemotherapy or radiotherapy.’ And then they stopped being friendly. Because I turned down that trajectory. (F1).*

Education and empowerment were considered important in order to maintain control and self-determination within health care. A respondent with breast cancer said:

*If I want to be the director of my health care, then I want to have moments of choice. I want to have information, and I want to be able to press a button to say ‘stop’. And, well, we don’t have that now, and there is a lot of work to do. I for instance would like to be present at my own multidisciplinary team meeting. But I can’t. (I2).*

Many of our respondents had experienced problems with access to standard of care, notably with referrals to the ‘right’ medical specialist. Though some patients had been adequately and rapidly referred to medical specialists such as dermatologists, cardiologists or oncologists in either regional or academic hospitals, others had less positive experiences with referrals. Patients were not always referred to tertiary care or expertise centers, when they themselves did deem this necessary. In the Netherlands, phase I clinical trials in oncology are generally offered only in university medical centers or specialized centers [15], to which physicians working in peripheral hospitals would have to refer. Respondents did not feel that doctors have financial incentives not to refer. Many doctors in the Netherlands are salaried employees and are not paid per patient or service offered. Rather, to our respondents, physicians’ hesitance to refer was best explained by professional pride:

*R: But doctors don’t even refer for trials in other hospitals. That’s where the problem starts […]. If doctors do not refer to opportunities outside their own hospitals, even though, for instance, there are trials available elsewhere, it they don’t even do that…*

*I: Do others too have the impression that doctors don’t refer to other centers?*

*R: Very many doctors don’t.*

*R. No, that happens very rarely.*

*R: I think doctors don’t do that. They all have a little bit of professional pride. And they want to fix [the patient] themselves. (FE).*

Expert patients believed that 50% of patients are actively counteracted by doctors when seeking referral care. Some patients reported that it took a long time for them to get a diagnosis, especially in cases of rare diseases. Patients felt that their doctors did not take seriously their complaints, and spoke of ‘diagnostic Odysseys’. A respondent with a rare disease said:

*On the other hand, because you know so much about [your own condition], you won’t let them send you away, and at the primary care physician it takes a very long time before you are taken seriously. […] You know, with primary care physicians, they are quick to think that something is wrong psychologically. You really have to say: and now I want you to refer me to someone. That I deplore sometimes. (F1).*

*I have had serious neurological problems since 2012, but I don’t know what is wrong. […] In the Netherlands it is very difficult to get diagnosed. You have to fight for years. And they want to send you to a psychologist first. It all goes very slowly, in fact, to come to a diagnosis. (F3).*

Respondents stressed that they had to take their treatment into their own hands. They were the ones that actively searched for treatment options when standard of care did not suffice. Often, they had to convince their doctors to try and use new possibilities. A patient who went abroad for a privately funded unapproved, investigational treatment said:

*R. The patients who are now being treated with [an investigational treatment], those are all patients for whom the question has come up from the inside, through the patient organization. And, well, I myself started this discussion, and I now know six, seven people who are all in this network, and I started this discussion with my doctor. And he will respond to [our wish to try this investigational treatment]… But he never brought it up himself. He never… He will not suggest something that he is not offering. (I3).*

#### The patient’s search for other options

Many of our respondents with chronic diseases actively participated in mapping out their treatment plan. Online, they searched for information about their condition and potential investigational treatment options. When they came across information about new treatments for their condition in newspapers or magazines, or on television, they pursued those leads and brought them up at their next doctor’s appointments.

Our respondents did not always distinguish between different types of investigational or ‘experimental’ treatments - between investigational drugs that are studied by physician-researchers in clinical trials and will eventually be evaluated for marketing authorization on the one hand, and ‘experimental’ treatments that are offered by complementary and alternative healers, or by quacks within the Netherlands or abroad, who are only pretending to be medical doctors, on the other hand.

*I felt like, before I go see a medical specialist, I’ll go on looking for other options, first. I thought, it won’t hurt to try. So I found a website of this natural doctor […] and they offered medications. So I thought: I’ll try it. So I went there. And they examined everything, and you start with a type of pill, one pill consisting of four small ones. And then you start to build up the dosage, building up and up. Until you observe change. (F2).*

Respondents were also looking for treatment options abroad that were not offered in the Netherlands. Patients went to Germany, Belgium, the United Kingdom and Turkey for diagnostic imaging, surgery, pharmaceutical drugs or other treatments.

Some respondents reported their experiences with the types of unapproved treatments we were looking for. One respondent spoke about an investigational agent for a chronic condition. This agent may have been approved for another condition (the respondent did not know), so this may have been an example of off-label use rather than pre-approval access:

*R. Did you swallow it or inject it?*

*R. No, you inject it, you inject it. I injected it instead of [another drug].*

*R. Right.*

*R. And I had discovered it myself, on the internet, because I was looking for something for someone else. I don’t have to look for myself any longer, I know that by now. But I thought: hey, this could be very interesting for me, too.*

*I. And how did your doctor respond to this?*

*R. Well, I requested a meeting with my pulmonary doctor. An in-between meeting. And well, it was an internist, actually. And he said: ‘Oh, how good that you found this out, because we are actually not quite there yet. But I think you deserve it, because you’ve done so much. Just to get yourself going again.’ And then I just got it. I had to come back every two weeks to get my blood tested, but it worked much better than [the other drug]. (F4).*

#### Reasons (not) to pursue non-standard treatment options

Respondents had very little personal experience with expanded access, neither with compassionate use programs nor with named-patient programs. In our focus group discussions, respondents tended to speak interchangeably about newly approved drugs, off-label use of existing drugs, and investigational treatments within or outside of the context of clinical trials. Patients sometimes could not tell the difference between scientifically sound and unsound ‘new’ or ‘experimental’ treatments. When we noticed this, we explained clearly that our study focused on drugs that were being studied by researchers in clinical trials. We asked respondents to imagine that a clinical trial would be ongoing in another European country, but not in the Netherlands, and that their treating physician could request permission to import the investigational drug through the named-patient program.

When asked under what conditions they would consider trying an unapproved, investigational treatment, respondents primarily mentioned conditions related to the scientific basis for the treatment: the treatment would have to be targeted at their particular disease, there must be ‘enough information’ about the mechanism of the drug, its possible side effects, and interactions with other drugs. One respondent indicated that the drug would have to have been tested in human beings, not merely in animals. Respondents wanted to know what the ‘measured effects’ of the investigational drug were on other patients, and what other patients’ subjective experiences were with the drug. One respondent said that she “would have to be in a group,” (F4) so that she knew that other patients, too, had considered the treatment and opted for trying. One respondent had heard about phases in clinical drug research, and about a time lag between marketing authorization and market availability, and indicated to want to use a drug during that time lag. Respondents were more willing to use unapproved drugs that had already been approved in other countries. A respondent with a chronic condition said:

*I wouldn’t swallow something if nothing is known about it. Then you’d really be a guinea pig. But I think that if it has been tested in another European Union country and if some results are available.. Then I assume, for European Union countries, that they are aligned more or less […] Then it won’t differ so much from the Netherlands, in terms of rules […] Then it seems safe to me. (F2).*

Secondly, respondents mentioned conditions related to the setting. Some respondents would prefer participation in a clinical trial over expanded access. For these respondents, there would have to be professional medical supervision and adequate monitoring; throughout the study, the doctor would have to be easily accessible, for instance through an emergency telephone number. One respondent said that a replacement should be available in case the treating physician fell ill. Also, the investigational treatment would have to be reimbursed. One respondent, who was talking about participation in a clinical trial, thought it was important that the drugs would remain available after completion of the trial: “Once I start it, I want to be given the chance, if I think it’s better, to keep it.” (F4).

Another important set of reasons for using investigational drugs was related to the health condition of patients: “if it could help me lead a normal life”, or “if I could be cured definitely, or at least if my symptoms could be alleviated.” (F1) Some indicated that with their current combinations of pharmaceutical treatments, they were doing all right, and they would only interfere with this precarious balance if their level of suffering increased: “If I can lead a minimally or reasonably normal life, I don’t need an investigational drug.” (F4) Others said that they would be interested in trying different, less painful or burdensome modes of administration for one or more medications they were using. For example, pills that could replace the (large) set of drugs that they were currently taking on a daily basis, or treatments that would have fewer adverse long-term effects or undesirable side effects.

Some said they would try investigational drugs - in or outside of clinical trials - without thinking, “because I have nothing to lose.” (F1) Respondents thought that patients who were suffering from fatal diseases or cancer were more likely to use investigational drugs than patients who were suffering from chronic (combinations of) conditions. Respondents generally agreed that the more despair they were experiencing, the more likely they would be to try investigational treatments.

*R. This sounds a bit weird, but the worse you’re doing, the more you would want to go for even the smallest chance.*

*R. The more you want to try. (F2).*

*Well, it won’t do much for me, because by now, I know from this internist that it cannot be cured, not with medications, not in any way. But in principle I would do anything to be cured, whatever it takes. I would start tonight. […] The pain that I know is going to come tonight when I go to bed… I will be screaming like a small child. Yes, well, that is a reason for me to say: I’d try anything. (F3).*

Apart from health reasons, respondents mentioned more altruistic reasons to participate in clinical studies: “to find fitting medications for myself and for others” (F2), for science, for “the future” (F3), for future generations of patients.

The burdens and risks associated with participation in clinical trials or usage of new drugs - whether approved or not (yet) approved - were mentioned as a main reason not to take part. Respondents were afraid of allergic reactions or complications, some because they had had (serious) adverse reactions to drugs in the past. Patients with chronic conditions said:

*Well, I didn’t do it. Because I think, well, it’s that exhaustion.. […] I didn’t feel up to it, because I would have to go to that hospital again and again, so I steered clear from that. (F1).*

*It should not be at the expense of my own health. You see, it shouldn’t like, affect my glucose levels, or cause me to get more hypo[glycaemia]s. It should not come at the cost of my health. I have become more and more careful about my health. (F2).*

Respondents were less willing to receive injections than to take pills, and less willing to take pills than to use creams. Also, respondents thought it was very important that their primary care physician or their treating medical specialist agreed with the investigational treatment. After all, it was felt, doctors had more knowledge of biology, health and disease, and they could point out possible adverse consequences. However, others said they would want to discuss it first with others - partners, children or parents - or make their own decisions before asking the doctor’s opinion.

One focus group participant, a young woman with metastasized cancer, narrated that she was “given up” (FE) in 2007, but - after some deliberation - decided to take part in a phase I clinical trial. Almost 10 years later, she was - inexplicably - still alive and using the drug, through a post-trial single-patient compassionate use program. Despite the life-saving effect that the drug had on her, it was no longer being studied for her condition, because of disappointing results in the wider group of research participants. When she was considering the option to take part in the study in 2007, what made her decide to enroll was the fact that she could always stop and withdraw whenever she wanted:

*You’ll get all sorts of information, also information to take home. I was talking it over with [my husband]. We were thinking: what are we going to do? And we decided, eventually, to just go for it, right? But the fact that I could stop at all times, that was the decisive factor. Like, if I’ll get to much discomfort at some point, I can always quit. (FE).*

The importance of being able to stop was stressed in several focus groups. One respondent mentioned that an ‘antidote’ should ideally be available.

One respondent with incurable cancer narrated how changes in his relationship with his son affected his perspective on his own treatment. He became more accepting of his nearing death, and less likely to try investigational treatments:

*Now I am much more relaxed than a year ago, when I was still alert and eager to find something that would give me more time to try and fix the problems that we [father and son] had together. And, well, so much calm has come into my life, just because these important issues have been solved. And there will always be other things, but I… I am done, completely done. Oh, I will go on until my tongue hangs out, as a manner of speaking. But with a good feeling. (I3).*

Some patients offered that it could be worthwhile, for patients who have “nothing to lose” (I1) to allow them to try investigational drugs earlier in the treatment trajectory, not only after all else has failed. When standard of care is associated with severe side effects and known not to be effective (e.g. combination chemotherapy for certain oncological conditions), patients may not find it acceptable to try standard of care first. In the early stages of disease, they prefer access to advanced treatments rather than having to first undergo conventional treatments of which the risk-benefit profile is better known, but not attractive. One respondent with incurable cancer recounted that his doctor had fought “by fire and sword” to prescribe an investigational or off-label treatment (the respondent did not know) before standard of care:

*We did it anyway. And it worked wonderfully well. My [tumor marker] dived downwards. And it held fantastically, for about a year. It was, well, super. (I3).*

#### Patients’ expectations of physicians with regard to non-standard treatment options

Patients whole-heartedly believed that it is the responsibility of treating physicians to inform their patients about the standard of care, but also about non-standard treatment options, including referrals to tertiary hospitals, clinical trials and opportunities for expanded access to investigational treatments, in the Netherlands or abroad. Respondents also assumed that doctors, especially those working in university medical centers or large (teaching) hospitals, were aware and had up-to-date knowledge of investigational treatments and clinical trials, around the world. Many respondents were aware of the existence of continuing education requirements for physicians in the Netherlands. Respondents recounted how they thought that physicians working in university medical centers attended scientific conferences, also abroad, and how they would learn about new treatment options and ‘what is in the pipeline’ (FE). Also, patients expected that doctors would propose investigational treatments that might be beneficial if these were available. When asked whether respondents expected their treating physicians to ‘look beyond’ for other options when standard of care was exhausted or insufficient, they said they did. It falls within the scope of physician’s tasks or duties, it was felt, to inform patients about treatment options that could potentially benefit them, including expanded access.

*R: I know from my quest on the internet, that there are actually studies for my tumor type. But my doctor has not mentioned them.*

*I: Did you expect that he would?*

*R: Yes, I guess I did. And to me that is a signal, right? A lot of work will have to be done to make sure that the patient gets all the information. (I5).*

*R. How far beyond is far beyond? […] There are specialists who are more like: ‘I really listen to my patient. My patient knows what he is feeling’, and they will respond to that. And that really makes a difference. Because you can go far beyond the basic treatment protocols.*

*R. That is a big difference.*

*R. I think he should go as far as the patient wants.*

*R. Yes.*

*R. Yes, it’s the patient who should decide.*

*R. Yes.*

*R. Yeah, well, sometimes, you have to protect patients, too. (F1).*

Others, too, said that doctors must sometimes protect their patients. When doctors have their doubts about a particular investigational treatment, it was felt by some respondents, they should not bring it up with their patients. Especially when treatments were potentially harmful or could be detrimental to patients’ psychological wellbeing, those patients need not be informed about such treatments. Respondents trusted that doctors would not offer them anything that they believed would pose unacceptable risks. Also, it was felt, doctors were allowed to offer their own views, and, for instance, actively discourage investigational treatments, when based upon adequate arguments.

*R. But how do you know whether a doctor knows what he’s talking about?*

*R. You don’t. (F2).*

Some respondents thought, however, that even if doctors have doubts about investigational treatments, they should start a discussion about these options:

*R. And this new doctor, she really brings everything to the table when I’m there, and she’ll inform me about these things.*

*I. And do you consider it to be her task, to do that?*

*R. Yes, yes, I do. Yes. Look, she..*

*R. I do, too.*

*R. She can totally say, I don’t believe in it, yet, or whatever, but she should bring it up.*

*R. Yes.*

*R. I do find that she should start the discussion. (F4).*

Some respondents narrated how they had discussed with their doctors, in advance, what would happen when the end of the standard treatment trajectory came in sight, and some doctors indicated that they were willing to resort to (further) non-standard treatment options when necessary. Respondents appreciated this.

*So we were talking about stopping this treatment, eventually. And she said: ‘I’m going to go see what we can do, right, things beyond the kinds of things that are going to be on the shelves by then. But when that time comes, right, I will go and look for you.’ So she really indicated, like, she would go out of her way… (FE).*

In spite of the information we provided, some respondents did not seem to understand the existing regulatory frameworks for expanded access, and believed that it is illegal. When asked whether respondents in one focus group thought that medical specialists should try and request expanded access for individual patients, they said:

*R. Yes.*

*R. Yes, if a patient wants to try something.. Give him a chance.*

*R. Yeah, well, I am doubtful whether the primary care physician, or any doctor, has any say in these matters.*

*R. I don’t think so.*

*R. I don’t think so either.*

*R. I think that he will be called to order by the government.*

*R. I think so, too. (F2).*

#### Reimbursement of non-standard treatment options

When patients have exhausted standard treatment options, it was felt by our expert patients, many doctors in the Netherlands do not propose non-standard treatment options. Reimbursement, or rather the lack thereof, according to expert patients, was one of the main reasons why doctors refrained from pursuing expanded access. One respondent talked about new surgical interventions:

*Well, these minimally invasive treatments, these I find a wonderful addition to the package [of standard treatment options] that we’ve already got. And what [we] have been working on, is saying: well, many of the oncologists will not mention this to patients, because one of the reasons why they won’t mention it, is that it is not covered by basic health insurance policies. (I4).*

Respondents felt that there are disparities among patient groups with regard to access to good-quality health care, and considered these unjust. Patients who are more assertive, health literate, affluent and/or resourceful could get access to treatments - or reimbursement of treatments - that others could not. One respondent told the group that although she did not have much money, she did have a big mouth:

*I have been very much at odds with my healthcare insurer, because these pills cost 365 euros or something, for two and a half months. And, well, I just cannot afford that. And eventually I acted on it, I wrote the House of Representatives, I wrote everybody. When I finally got [a regional television network] involved, and, well, the same day [the insurance company] gave me my medicines back. (F1).*

Respondents were generally very willing to pay for investigational treatments, if they had to. Even if the investigational treatment were very expensive, they would try and find alternative means to obtain the drug.

*R. If I could get something illegally, or buy something, that would really work, well, I’d sell something, so that I could make sure that I’d be able to buy it.*

*R. Yes, I agree.*

*R. Me, too. I’d find a way.*

*[…]*

*I. But what if it would be very expensive?*

*R. I’d still do it.*

*R. Would you?*

*R. Yes. […].*

*R. But don’t you think that the healthcare insurer should pay for it?*

*R. I do, actually. But they won’t. (F2).*

A respondent who was disabled, alone and suffering from terrible pains, explains:

*My daughter, she has a very important position, she informed in [a far-away country], when I was just diagnosed. And they have a machine there, and you go in for three weeks. And it purifies the blood or something, anyway, when you come out, you’re perfectly healthy. But it costs 150,000 euros. […] So that is the story. I could have been cured, but I didn’t have the money. And now I’ve been stuck with this for ten years or so. (F3).*

Expert patients thought it was unethical to have patients pay for investigational treatments themselves. They thought that health insurers or the state should reimburse investigational treatments. One patient told us about an investigational treatment that he had been given abroad, which is four times less expensive than standard of care for his condition in the Netherlands, and, according to the respondent, more effective (I1). While his healthcare insurer supported future inclusion of the investigational treatment in the Dutch national basic healthcare insurance package in lieu of (much more expensive) existing standard of care, it was not willing to reimburse the costs of this patient’s treatment abroad.

Patients who had gone abroad to try investigational treatments reported that the expenses were worthwhile, that they benefited somehow, or for some time, and did not regret their decisions. But these decisions clearly did come at a cost:

*Look, it has cost me 20,000 euros, and it has been worth it. So now I’ve sold my house. Because I’m thinking: well, if I can get a treatment somewhere that I’ll have to pay for myself, I want to have money in the bank. And my children say it, too: ‘We’d rather have a living mother in a rental apartment than a dead mother in a privately owned house.’ (I1).*

Another respondent had the means to go to a nearby country to try an investigational treatment, but acknowledged - and found problematic - that many other patients with the same condition were not able to afford such medical travel.

*I am in the lucky position that I can afford doing crazy things, doing these things on my own initiative, financially. This [investigational procedure] costs 6000 euros per treatment. […] But now it’s time for the next step. I am working with [a hospital in the Netherlands] to raise funds, because they are looking for fifty or so research participants [to validate the treatment], and they don’t have any budget. And the painful thing about this situation is, I get phone calls from one or two people every day, who have read [an interview with this patient about this treatment abroad], who want to know more about it. […] I think it is unacceptable, in fact, that this happens, that I, incidentally, can afford this, while all of the others cannot. I don’t feel good about this. […] You want these things to be accessible for everybody. (I3).*

## Discussion

This qualitative study was the first to provide insight into what patients with unmet medical needs want with regard to unapproved, investigational treatment options, and what they expect from their treating physicians. Our findings show that patients with unmet medical needs in the Netherlands have very limited knowledge about and experience with expanded access. However, many – but not all – of our respondents were well informed and ‘empowered’ with regard to their own health condition and medical care. Some patients were very knowledgeable about their conditions and/or active in their respective patient communities, which may have led them to have different experiences and views than patients who were less informed and/or less active. The idea that patients must be assertive in order to get access to best available care was widely expressed and supported by our respondents. Based on our findings, five areas of ethical concern can be discerned.

First, when patients have exhausted standard treatment options, they may start looking for other non-standard options. Patients are finding their way to open access publications of clinical trials on the internet, and especially some of those who suffer from rare, chronic or long-term progressive disorders are becoming knowledgeable about their conditions [19], about standard of care, and about ongoing clinical trials in their own country or abroad. However, this does not apply to the majority of patients. Many of our respondents had little knowledge of systems for drug development, clinical trials, marketing approval by drug regulatory authorities and/or health technology assessment and reimbursement policies. Some respondents were unable to distinguish between new drug treatments that were under investigation by healthcare professionals in academic hospitals on the one hand, and ‘experimental’ complementary or alternative treatments on the other hand. Patients often did not know whether the drugs they took were approved for their indication, for other indications (off-label use), or not at all. It has been observed that expanded access, so far, has remained limited to patients who were well-off and/or influential and whose partners or close friends were expert medical specialists [1, 3, 20]. Our study confirms the existence of disparities along the lines of health literacy and connectedness, raising concerns with regard to equal access to non-standard treatment options.

Second, our respondents stressed that decisions with regard to (non-standard) treatment were ‘highly personal’ in nature. Patients were not unequivocally willing to try new drugs. Rather, they had nuanced views with regard to investigational drugs, explaining their willingness to take part in expanded access in terms of factors related to the scientific evidence of the safety and efficacy of the drug (e.g. respondents were hesitant about first-in-human studies) and factors related to the organization of health care surrounding the use of an investigational drug (e.g. the treating physician would need to be involved; they would need to be monitored closely). Also, many patients with chronic conditions were afraid to disturb what they perceived to be a precious equilibrium that they were able to maintain with their current combinations of drugs and lifestyle adjustments, and as long as they felt ‘okay’, they were reluctant to try something new. This is consistent with earlier reports on oncology patients in the Netherlands, for instance, who refused to participate in phase I trials because of low expectations of benefit, concerns about side effects, and their negative effects on the patient’s current - and, for the time, acceptable - condition [15]. However, patients with a life-threatening disease who had run out of standard treatment options and were suffering from refractory symptoms, were less hesitant to try investigational drugs. Patients with chronic conditions and patients with life-threatening conditions thus seemed to differ in their readiness to try investigational drugs. The fewer patients’ options and the greater their despair, the greater was their willingness to pursue the use of non-standard treatment options.

Third, patients volunteered many stories about their general experiences with medical specialists. Although some patients reported negative experiences with medical specialists, most had high levels of trust in their doctors and high expectations of their doctors. Patients expected, for instance, that doctors are well aware of scientific developments in their fields, and of new treatments being studied around the world. They believed that doctors would inform them about all treatment options, including those that are not approved for marketing and still under investigation, and including those that are available only in other hospitals or even countries. They expected that doctors would actively look for non-standard treatment options when standard treatment options were exhausted, and, importantly, they considered it to be a doctor’s duty to do so. Some respondents indicated that they would try investigational drugs only if their doctor recommended and/or supported it. In spite of prevailing ideals of patients’ self-management and shared decision-making, doctors continue to have a decisive role in determining their patients’ treatments. It is important to note that contrary to patients’ expectations, doctors do not always mention clinical trials [21], and, as our study indicates, will rarely mention existing opportunities for expanded access.

Fourth, patients with chronic conditions and/or unmet medical needs showed a high willingness to pay for unapproved, investigational drugs. Few respondents said that they had ethical problems with having to pay out of pocket for non-standard treatment options: it was felt that one’s health is so important that it trumps other priorities, and that one will have to find other ways to fund the treatment, such as crowd funding. More and more patients resort to online petitions and social media campaigns [22], raising ethical concerns [23]; campaigns may not always succeed, and success may not be fairly distributed among patients. Even if patients succeed in amassing the funds, private money does not always buy them drugs. In most hospitals in the Netherlands, it is generally not considered morally acceptable to receive out-of-pocket payments from patients for ‘extra’ (non-standard) health services; in most cases, either the health insurer agrees to reimburse the treatment (out of leniency), or the treatment is simply not provided. Health insurers will typically not reimburse investigational drugs, because scientific evidence of its effectiveness is often lacking. Thus, investigational drugs would need to be supplied by the pharmaceutical company at no cost, ‘out of compassion’. Companies, however, may not always be willing or able to do so. Thus, as the options in the Netherlands may be limited, some Dutch patients are using private or otherwise collected funds (e.g. crowd funding) to seek new treatment options abroad [24].

Either way, respondents in our expert patient focus group found it unacceptable to let patients pay themselves. They felt that the development of a transparent and equal funding system for expanded access should be a priority for policy makers. For instance, it was suggested, governments, health insurers and drug developers could each contribute to a fund dedicated to cover the costs of investigational drugs for patients who are eligible for expanded access. In the international literature on expanded access, alternative funding arrangements are being considered, including the setting up of foundations or non-profit organizations as intermediaries [25].

Fifth and finally, our findings suggest that although expanded access may benefit patients (one respondent was kept alive by an investigational drug), it may also harm them, in various ways. One of the most prominent ethical issues in the literature on expanded access is the question of false hope [26, 27]. When the chance at medical benefit is very slim, will not the sheer existence of opportunities for expanded access lead to false hope among patients who are dying? One interviewee narrated how she had sold her house in pursuit of a cure, to fund trips abroad for investigational treatments. This patient may spend all her money on treatments that are not likely to take away her illness. She was still alive, however, in spite of her Dutch treating physician’s expectations. Our respondents seemed to understand that there are risks associated with the use of an investigational agent, the safety and efficacy of which will not have been fully established. Some of these risks are unknown and unquantifiable, which makes it difficult to make informed decisions whether or not to proceed with requesting access. When asked how they would make such decisions, respondents with chronic conditions were well aware of everything they stood to lose, were careful to avoid side effects, and not to needlessly imperil a precarious health equilibrium or an acceptable quality of life. Respondents said that in the face of life-threatening illness, however, they would take more risks and be more willing try an investigational drug with a small chance at medical benefit. Thus, patients who are facing death may be more vulnerable to high hopes and (financial) exploitation.

In the future, ethical discussions on expanded access should focus on three areas: first, strengthening of decision-making and informed consent processes by patients with life-threatening disease, and on the blurry line between hope and false hope; second, addressing the disparities in access to investigational treatments across patients groups of varying levels of health literacy and assertiveness; and third, addressing the gap between patients’ expectations of their treating physicians and the existing reality in which doctors may have limited awareness of opportunities for expanded access and may not recognize a ‘duty’ to provide information about expanded access to patients who run out of standard treatment options.

### Limitations

While we found expert patients to be highly motivated to take part in our qualitative study, it was difficult to recruit respondents who were more representative of the Dutch general population of patients with chronic conditions or unmet medical needs. With the help of a recruitment agency, we were able to engage respondents who were diverse in terms of age, gender, educational background and health literacy. Still, our respondents were rather assertive with regard to their health, and may have differed in this respect from other Dutch patients. Studies have found, for instance, that most patients are not actively looking for non-standard treatment options such as clinical trials, and tend to follow recommendations by their doctors instead [21, 28, 29]. Our respondents were generally less passive, and more actively involved in searching for and decision-making regarding treatment options. Concerns regarding the introduction of bias through the engagement of atypical patients in research have been documented [30], and may limit the generalizability of our findings. Further empirical research efforts may need to focus on mapping patients’ experiences and views across different patients groups and disease groups. Patients may differ in level of understanding, but also in terms of relevant personality traits, such as willingness to take risks, which may lead them to have different needs regarding, for instance, information provision about expanded access.

Moreover, at the outset of our research project, we meant to include patients who were running out of standard treatment options and considering the use of investigational drugs, or patients who had used investigational treatments in the past. However, such patients were difficult, if not impossible, to find, given the very low uptake of opportunities for expanded access in the Netherlands. While we did talk informally to patients who had ALS or metastasized melanoma, we did not want to recruit them for audiotaped interviews or focus groups, because they were very ill and potentially life-saving treatment options were at the time not available to them.

In addition, there are particularities of the Dutch health care system, such as the relative infrequency of malpractice litigation, the ‘conservative medicine’ attitude of primary care physicians, aimed at avoiding over-diagnosis and overtreatment (although Dutch general practitioners report that they satisfy patients’ demands for referral too easily, resulting in patients receiving ‘too much’ care in hospitals) [31], and the system of universal health coverage and mandatory health insurance, which may limit the external validity of our study. Policy discussions on expanded access would thus benefit from further qualitative studies of patients’ experiences conducted in other countries.

Finally, many of the ethical issues raised, including informed consent, the risk of exploitation, the notion of false hope, potential medical and psychosocial harms, funding issues and equal access, merit further normative scrutiny.

## Conclusion

Our respondents, Dutch patients suffering from a range of serious, chronic, rare, progressive and/or life-threatening diseases, were assertive and actively looking for other non-standard treatment options. Nevertheless, they had very little knowledge about and personal experience with unapproved, investigational drugs outside the context of clinical trials. When asked under what conditions they would try investigational treatments, they mentioned highly personal considerations ranging from the level of available evidence to the active involvement of their treating physician, and from potential interactions with their current medications to the mode of administration. Patients were generally protective of their quality of life and current management of their medical condition(s), and would try investigational drugs only when they suffered, ran out of options, and had little left to lose. These findings strengthen the notion that (at least some) patients with chronic conditions are capable of making well-considered choices with regard to the use of investigational drugs. Patients with life-threatening illness, however, are more willing to take risks and try investigational drugs, even in the context of a small chance at medical benefit. Also, when patients are confronting death, they are more willing to spend private money on investigational drugs. In this patient group, the blurry line between hope (against a small chance at medical benefit) and ‘false hope’ should be studied and addressed.

Over the next couple of years, physicians, pharmaceutical companies, and policy-makers will be shaping policies and practices of expanded access. In doing so, they should be responsive to patients’ views and concerns. Patients might expect their treating physicians to inform them about investigational treatments when the end of a standard treatment trajectory comes into view, even if their chances at cure, prolongation of life, or improvement of quality of life are slim. Also, patients with unmet medical needs - especially those with life-threatening disease - might be willing to pay a high price for a small chance at medical benefit, which might render them vulnerable to exploitation. This concern might especially be relevant to healthcare systems in which fair and transparent funding arrangements are lacking for expanded access.

## Supplementary information

**Additional file 1.** Interview Guide, Interview guide for focus groups and individual interviews with patients (translated to English by EB).

## Data Availability

Transcripts of the interviews and focus groups are in Dutch and are not published to protect the privacy of respondents. Transcripts can be made available upon request by the researchers.

## Abbreviations

EMA: European Medicines Agency
FDA: Food and Drug Administration
NFK: Dutch Federation of Cancer Patients
F1–4/FE: Focus group 1–4/Focus group with expert patients
I1–5: Interviews 1–5

## Footnotes

1 When we use the term ‘standard treatment options’ in this paper, we are referring to treatment options that are curative or life prolonging and that are part of standard of care and/or incorporated in clinical guidelines for specific diseases or disorders. Palliative treatment options can also be part of standard of care and/or incorporated in clinical guidelines, but is aimed at the improvement of quality of life, not at a cure. We take the term ‘non-standard treatment options’ to refer to treatment options that are off-label, not (yet) approved for marketing, investigational, and being studied in clinical trials or prescribed within expanded access programs, including named-patient and compassionate use programs. They are not part of standard of care and are not (yet) incorporated in clinical guidelines in the Netherlands.

2 A ‘valorization panel’ consists of stakeholders who offer feedback on the research questions, approach and results, and assist with the dissemination of the research results. Our valorization panel included representatives of patient organizations, physicians’ organizations, (hospital-based) pharmacists’ associations, trade associations for the pharmaceutical industry, and regulatory and health technology assessment authorities. The set-up of a valorization panel is a requirement of the Responsible Innovation program at the Netherlands Organisation for Scientific Research, which funded the research project (https://www.nwo-mvi.nl/showcase/value-valorisation-panel). The valorization panel serves as a counterweight to the collaboration with one or more private partners (in our case, myTomorrows), another central requirement of the Responsible Innovation program.
