# Supplementary material for: What do patients with unmet medical needs want? A qualitative study of patients’ views and experiences with expanded access to unapproved, investigational treatments in the Netherlands
Source: BMC Med Ethics. 2019 Nov 9;20:80. doi: 10.1186/s12910-019-0420-8 (PMC6842468; doi:10.1186/s12910-019-0420-8)
Supplement: Supplementary file 1 — Additional file 1. Interview Guide, Interview guide for focus groups and individual interviews with patients (translated to English by EB). [file 12910_2019_420_MOESM1_ESM.docx]

**Interview guide focus groups and interviews *Nice to meet?* project**

- **Themes**
- Standard treatment options, off-label use of medicines, second opinion, referral to tertiary or academic medical centre, clinical trials, use of investigational drugs outside of clinical trials.
- Access to investigational medicines
  - Decision-making and considerations of the patient
  - Role of the treating physician
  - Knowledge of existing options for access to investigational treatments
  - Actual experience with use of investigational products
  - Funding
  - Other non-standard treatment options (inter alia abroad)
  - Information provision (physicians, media, google, patient organisations, clinicaltrials.gov)
- **Contents and timeline focus groups**

Introduction (10-15 min)

- Thank you for taking part in the discussion
- Brief introduction of ourselves
- Brief introduction of the research project
  - Developments in the area of ‘early access’
  - Ethical concerns
  - Role of patients and physicians
  - Approach: interviews/survey/focus groups in the Netherlands, Turkey and the United States of America
- Explanation of the aim of the focus group discussion
  - Why have you been invited? We invited patients with various types of conditions (inter alia neurological, oncological and metabolic/endocrine conditions), for whom investigational treatments might be relevant, as effective standard treatment options might be either absent altogether or exhausted at some point.
  - Aims: to map the experiences, interests and concerns of patients for whom the use of unapproved, investigational drugs might at some point become an option. What do patients know about existing opportunities to access investigational drugs? What are their views on the use of unapproved, investigational products? What are their experiences? What are important considerations for them to decide to use or not to use investigational treatment options? What do patients expect from their doctors in relation to ‘early access’? With these focus group discussions, we are hoping to gain better insight in the needs and concerns of patients who may be confronted with such options in practice, also with an eye to the future.
  - For ourselves: the aim, for all focus groups, is to have the participants talk spontaneously as much as possible about the themes presented by us, in their own words, and to have them respond to each other. If participants do not mention certain aspects (that we had expected they would), this could also be a relevant finding. This means that we let the discussions flow naturally, and intervene as little as possible.
- Explanation set-up focus group:
  - This is a safe environment. Everyone can speak freely. There are no good or bad answers. We are interested in your views and experiences. Your views and experiences count.
  - Confidentiality and anonymity. Why do we audio-tape this discussion? We will transcribe the audiotape and use the transcripts for analysis. Your name and personally identifying information will not be used in publications. The tapes and transcripts will be safely stored at Erasmus MC.
  - For transcription purposes, it is important that we speak taking turns and not together.
  - After the focus group we will send you a report of the discussion.

Introductory round (30 min)

Name, age, and, if you would like, something about your professional life, things you like to do in your free time, or where you are from, and: your medical condition and history.

Themes

- Standard treatment options, second opinion, referral to tertiary of academic medical centre (40 min)

Most patients are treated according to a standardized protocol. For many oncological conditions, for instance, the preferred option is surgery, and additional radiotherapy and/or chemotherapy. Medical treatments are offered to patients in a specific order. When several lines of treatments have been tried and failed, it may happen that there are no further standard treatment options available. For some conditions, there are no effective treatment options at all, for instance in rare metabolic disease or in ALS. Are you aware of the standard treatment protocol for your condition? What are your treatment options? And in what order will they be offered to you? Has your doctor explained this to you?

For some conditions, there are special centres of expertise in the Netherlands, for instance in university medical centres, where doctors may be specialized in certain conditions. Some patients are referred to such centres, if they can be treated better there than elsewhere. Have you been referred to another centre? How did this happen? On whose initiative did this happen? Would you expect your doctor to propose a referral when relevant? Have you ever requested a second opinion?

- Access to investigational treatment options (40 min)

Before a new drug can be marketed, a lot of research must be done. This may take years. The research usually consists of multiple phases of clinical trials, in which safety and efficacy are tested on human research participants. During this development phase, some patients may use the drug as research participant, and, in rarer cases, outside of the clinical trial. This applies to drugs that are seen as very promising. At the same time, little is known at that point about the safety and efficacy of the drug. It is not clear whether the expected positive effects of the drug will outweigh side effects and risks.

Would you wish to consider the use of an unapproved, investigational drug? Are there conditions under which you would or would *not* consider such use? What are important considerations? Please write down for yourself first, and afterwards, we will discuss as a group. […]

What is or should be the role of the doctor? What do you expect from your doctor in relation to information provision about investigational drugs? May the doctor bring up the subject? Should the doctor bring up the subject? Do you believe that it is a doctor’s task to ‘look around’ for investigational treatment options for patients who have run out of standard treatment options? Should doctors actively look for non-standard treatment options? Or should they not?

Some people (including doctors) are against the use of investigational treatments outside of clinical trials, inter alia because of the limited information about the efficacy of the drug. It would amount to giving patients ‘false hope’. Another argument is that the use of medical drugs is often associated with side effects, some of which may be serious and dangerous. Should patients be protected against false hope and potential side effects? What would that protection look like? Should investigational treatment options be banned, or not-offered, or should doctors not inform patients about such options?

Patients who cannot be enrolled in clinical trials can sometimes use an investigational treatment directly (through *named patient* or *compassionate use* programs). Doctors can apply for this. In practice this does not happen often, because doctors do not know about or support the possibility, because they are not convinced that the benefits may outweigh the risks, of because the pharmaceutical company cannot or does not want to supply the drug. Also, there are no funding arrangements for such ‘early access’ programs in the Netherlands, and so it is unclear who should pay for this type of treatment. Have you heard of these programs? If yes, could you tell us about your experiences with these programs?

Funding of expensive newly approved drugs is a recurring topic of societal discussion at the moment. Unapproved, investigational drugs are not reimbursed, because they do not (yet) belong to the ‘state of science and practice’. What are your views about this? Should health insurers pay for the use of investigational products? Should pharmaceutical companies pay for the costs? Or hospitals? Or should governments set up a special fund?

Could you tell us about your experiences with travelling abroad for non-standard treatment options that are not made available in the Netherlands? If you are aware of such non-standard treatment options, how have you learned about these options? From the internet, from patient organisations, from you doctor?

- End of the discussion (10 min)

The moderator offers a summary of the most important aspects discussed during the focus group discussion. Participants are given the opportunity to respond to the summary and to ask questions. The moderator explains again what will happen with the audiotape. Participants are given a small present as a compensation for their time and efforts. If they like, they can provide their email addresses to receive information from us on the proceedings of the research project.
